# Adverse Events Following Cow’s Milk-Derived Human Milk Fortification: A Nationwide Human Milk Bank Study

**DOI:** 10.3390/nu18172755

**Published:** 2026-08-23

**Authors:** Yuki Tani, Katsumi Mizuno

**Affiliations:** 1 Department of Pediatrics, Showa Medical University, Tokyo 142-8666, Japan; yukitani1330@naramed-u.ac.jp; 2 Department of Pediatrics, Nara Medical University, Kashihara 634-8522, Japan

**Keywords:** human milk fortification, donor human milk, cow’s milk-derived fortifier

## Abstract

**Background:** Cow’s milk-derived fortifier (CMDF) is widely used to improve the nutritional adequacy of human milk for very preterm infants. Although generally well-tolerated, feeding difficulties may occur during CMDF use. Characterizing these events and their associated clinical factors may improve the understanding of feeding tolerance in routine neonatal practice. **Objective:** To characterize documented adverse feeding events among infants receiving donor human milk (DHM) and CMDF and to identify clinical characteristics associated with these events. **Methods:** This retrospective nationwide cohort study used the Japanese Human Milk Bank Database. Infants receiving DHM and CMDF were included. Clinical characteristics were compared between infants with and without documented adverse feeding events, and multivariable logistic regression was used to evaluate associated factors. **Results:** Among the 2587 infants receiving DHM and CMDF, 2476 had available adverse-event information. Adverse feeding events were documented in 315 infants (12.7%), most commonly abdominal distension, increased gastric residuals, vomiting, and suspected cow’s milk protein allergy. Infants with documented adverse feeding events had lower gestational age and birth weight and required longer to reach an enteral feeding volume of 100 mL/kg/day. In multivariable analysis, delayed achievement of 100 mL/kg/day remained associated with documented adverse feeding events, whereas gestational age, birth weight, and sex were not independently associated. Routine clinical variables showed limited discriminatory ability (AUC 0.611). **Conclusions:** Most infants receiving CMDF had no documented adverse feeding events. Slower progression to an enteral feeding volume of 100 mL/kg/day was associated with documented adverse feeding events; however, the temporal relationship among feeding progression, CMDF initiation, and adverse events could not be established. These findings should therefore be considered hypothesis-generating rather than predictive and require prospective validation.

## 1. Introduction

Human milk is the recommended enteral nutrition for very preterm infants because it is associated with reduced risks of major neonatal morbidities, including necrotizing enterocolitis (NEC), late-onset sepsis, bronchopulmonary dysplasia, and retinopathy of prematurity [1]. When mothers’ own milk (MOM) is unavailable or insufficient, pasteurized donor human milk (DHM) is recommended as the preferred alternative for high-risk infants [2].

However, although human milk provides numerous bioactive and immunological components that are not available in formula, unfortified human milk alone does not fully meet the nutritional requirements of very preterm infants. Even preterm MOM may provide insufficient amounts of protein, calcium, phosphorus, and other nutrients required to support optimal postnatal growth [3]. In addition, DHM generally contains less protein than preterm MOM, partly because donor milk is often obtained at later stages of lactation [4]. Consequently, human milk fortification is an essential component of nutritional management for very preterm and very-low-birth-weight infants [3,5].

This issue is clinically important because a large proportion of very preterm infants cared for in neonatal intensive care units require fortification during hospitalization. Globally, an estimated 13.4 million infants were born preterm in 2020, accounting for approximately 9.9% of all live births [6]. Early nutritional management in this vulnerable population is closely related to postnatal growth, establishment of full enteral feeding, and subsequent neurodevelopmental outcomes [7,8,9,10,11]. Therefore, even relatively uncommon feeding-related adverse events following fortification may affect a clinically meaningful number of infants, delay advancement of enteral nutrition, and complicate nutritional management.

Cow’s milk-derived human milk fortifiers (CMDFs) are widely used to increase protein and mineral intake and support postnatal growth. Although generally well-tolerated, feeding intolerance and gastrointestinal adverse events have been reported in some infants receiving CMDF [12,13,14,15,16,17,18]. Importantly, human milk fortification should not be regarded as competing with breastfeeding or the provision of MOM. Rather, fortification enables the continued use of human milk while supplying the additional protein, minerals, and micronutrients required for adequate growth in very preterm infants [1,3,5].

The choice of fortification strategy also has health-system implications. Human milk-derived fortifiers permit maintenance of an exclusive human milk diet (EHMD) but may be constrained by product availability, donor milk supply, cost, reimbursement, and institutional resources. Consequently, access may vary among institutions and healthcare systems [19,20]. Previous studies comparing EHMDs with diets containing bovine-derived products have primarily focused on NEC and other major neonatal morbidities, whereas considerably less is known about the clinical characteristics associated with documented adverse feeding events among infants receiving CMDF in routine neonatal practice. Better characterization of feeding tolerance during CMDF use may therefore help inform future prospective studies of individualized fortification strategies and more equitable use of limited healthcare resources.

Therefore, the aim of this nationwide study was to characterize documented adverse feeding events among infants receiving DHM and CMDF and to identify clinical characteristics associated with these events. We further explored the associations between routinely available clinical characteristics and documented adverse feeding events to generate hypotheses for future prospective studies of individualized fortification strategies.

## 2. Methods

Clinical information was entered into the database by healthcare professionals at participating NICUs using standardized data fields. Adverse feeding events were recorded according to predefined categories. More than one adverse-event category could be recorded for an individual infant when multiple symptoms were observed. For the primary analysis, however, the unit of analysis was the infant rather than the individual adverse event; therefore, an infant with one or more reported adverse feeding events was classified once in the adverse-event group. Individual adverse-event categories were analyzed descriptively and were not treated as independent observations.

### 2.1. Study Design and Data Source

This retrospective cohort study was conducted using the nationwide Japanese Human Milk Bank Database, which prospectively collects clinical information on infants receiving DHM. The database includes demographic characteristics, nutritional management, clinical outcomes, and adverse events. Data collected between February 2018 and June 2026 were included in the present study.

Clinical data were entered by healthcare professionals at participating NICUs using structured database fields. The database was designed for longitudinal recording at the individual-infant level, thereby allowing nutritional characteristics and clinical events to be linked to each infant without treating repeated events as separate patients. Before analysis, the dataset was reviewed for completeness, internal consistency, and implausible values using the same quality-assurance procedures applied in our previous database studies.

### 2.2. Study Population

Infants were eligible if they (1) received DHM and (2) subsequently received CMDF. The primary comparative analysis was restricted to infants with available information on documented adverse feeding events. Infants lacking information on the primary outcome were excluded from the primary comparative analysis. Multiple imputation was not performed for missing adverse-event status because the missing variable represented the primary outcome itself rather than a predictor or covariate, and the assumptions required to reliably impute the presence or absence of an adverse feeding event could not be verified from the available database information. Missing values for individual covariates were handled by available-case analysis for the relevant comparisons and regression models.

### 2.3. Nutritional Management

All infants received DHM according to each NICU’s standard feeding protocol. The exact timing, dose, and formulation of CMDF initiation were not available in the database, and institution-specific feeding and fortification protocols were not systematically recorded. Therefore, the day on which enteral feeding reached 100 mL/kg/day was evaluated only as a pragmatic measure of enteral feeding progression. This variable was not intended to represent the timing of CMDF initiation or a validated measure of gastrointestinal maturation.

### 2.4. Outcomes

The primary outcome was the presence of documented adverse feeding events among infants recorded as having received CMDF. Adverse feeding events were recorded in the database using predefined categorical fields, including abdominal distension, increased gastric residuals, vomiting, bloody stool, suspected cow’s milk protein allergy, NEC, and other gastrointestinal symptoms. NEC was recorded as stage 2 or higher; however, detailed operational definitions and severity criteria were not uniformly available for several other adverse feeding events, such as quantitative thresholds for gastric residuals or abdominal distension, or whether blood in the stool was occult or gross. These events were therefore analyzed as clinician-reported adverse feeding events rather than centrally adjudicated diagnoses.

More than one adverse-event category could be recorded for an individual infant when multiple manifestations were observed. For the primary analysis, however, the unit of analysis was the individual infant rather than the individual adverse event. Thus, an infant with one or more documented adverse feeding events was classified once in the adverse-event group, whereas infants with no documented adverse feeding events were classified in the non-adverse-event group. Individual adverse-event categories were summarized descriptively and were not treated as statistically independent observations. Accordingly, the sum of individual event categories could exceed the total number of infants with adverse feeding events.

### 2.5. Variables

Baseline variables included gestational age, birth weight, sex, and the day on which an enteral feeding volume of 100 mL/kg/day was achieved. These variables were selected a priori on the basis of clinical relevance and consistent availability in the nationwide database. Gestational age and birth weight were included as markers of biological immaturity. Sex was included because previous large-scale neonatal studies have reported sex-related differences in morbidity among very preterm infants [21]. The day on which enteral feeding reached 100 mL/kg/day was included as a pragmatic measure of enteral feeding progression and was not considered a validated marker of gastrointestinal maturation or a proxy for the timing of CMDF initiation.

Other potentially relevant factors, including detailed respiratory status, medication exposure, the exact timing and dose of fortifier initiation, and institution-specific feeding protocols, were not consistently available or sufficiently standardized in the database and therefore could not be included in the present analysis.

### 2.6. Statistical Analysis

Because the study period extended from 2018 through 2026 and included the COVID-19 pandemic, calendar year was additionally evaluated as a potential temporal confounder in a sensitivity analysis. The multivariable model was re-estimated after adjustment for calendar year to determine whether temporal changes in neonatal care or feeding practices materially affected the observed associations.

Continuous variables were compared using the Student’s *t*-test or the Mann–Whitney *U* test, as appropriate. Categorical variables were compared using the χ^2^ test or Fisher’s exact test. Variables considered clinically relevant, including gestational age, birth weight, sex, and the day on which enteral feeding reached 100 mL/kg/day, were entered into a multivariable logistic regression model to identify independent factors associated with documented adverse feeding events. Receiver operating characteristic (ROC) curve analysis was performed to evaluate the discriminatory ability of routinely available clinical variables.

This study was approved by the Institutional Review Board of Showa Medical University (Approval No. 21-084-A).

## 3. Results

### 3.1. Study Population (Table 1)

A total of 2587 infants received donor human milk followed by bovine-derived human milk fortification during the study period. Of these, 2476 infants had available information regarding documented adverse feeding events and were included in the primary analysis. Documented adverse feeding events were identified in 315 infants (12.7%), whereas 2161 infants (87.3%) had no documented adverse feeding events (Table 1).

**Table 1:** Characteristics of the study population.

Characteristic	Value
CMDF-exposed infants, n	2587
Gestational age, weeks, median (IQR)	28.4 (26.1–30.7)
Birth weight, g, median (IQR)	982 (728–1259)
Infants with available AE information, n	2476
Adverse feeding events, n	315
No adverse feeding events, n	2161
Missing AE information, n	111
AE rate among infants with available AE information, %	12.7

Gestational age was available for 2580 out of 2587 infants. AE, adverse feeding event; CMDF, cow’s milk-derived fortifier; IQR, interquartile range.

### 3.2. Baseline Characteristics

Infants with documented adverse feeding events had significantly lower gestational age and birth weight than infants without documented adverse feeding events. They also required a significantly longer time to achieve an enteral feeding volume of 100 mL/kg/day. No significant difference in sex distribution was observed between the two groups (Table 2).

### 3.3. Types of Adverse Feeding Events

**Figure 1:**
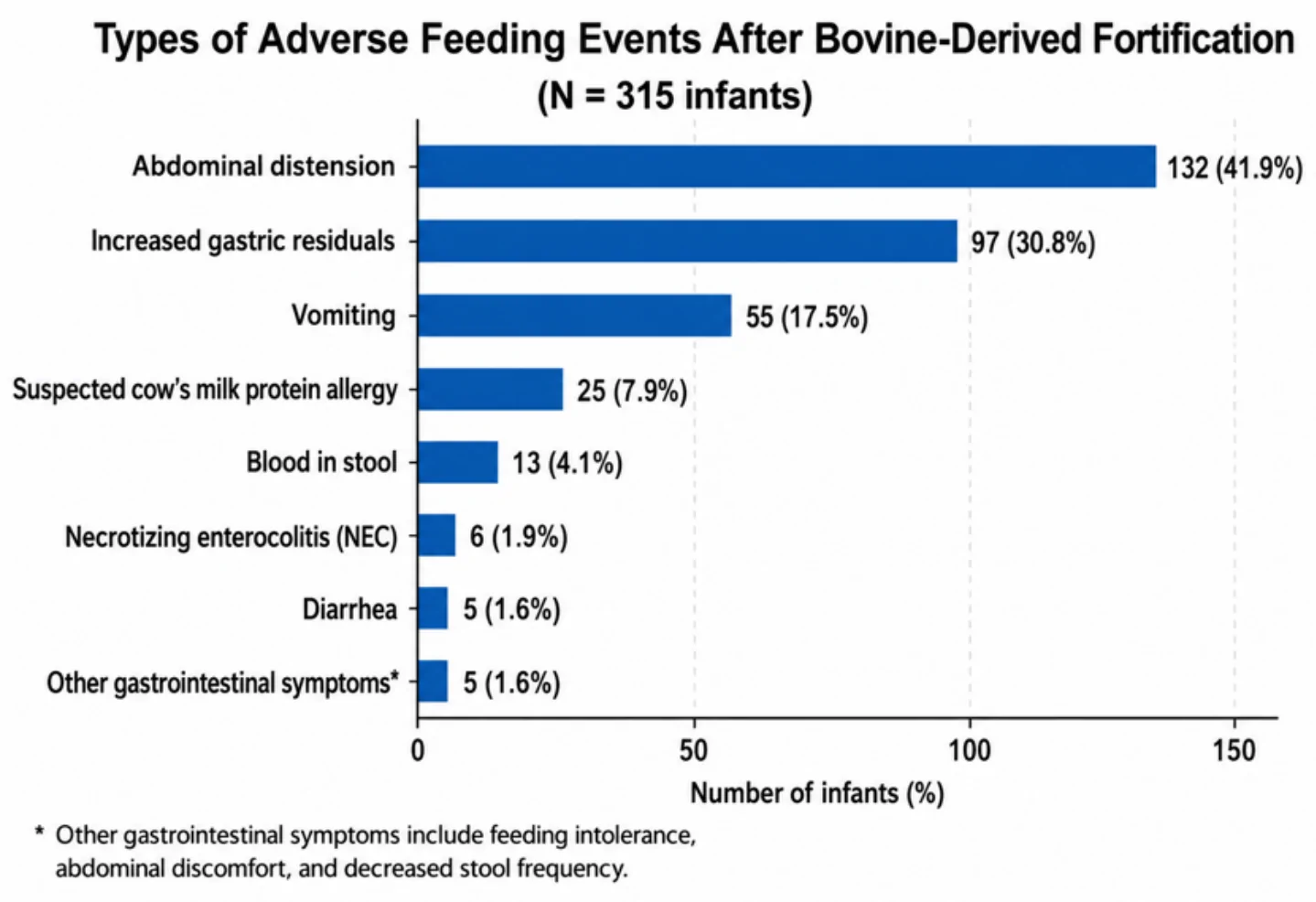
Types of documented adverse feeding events among infants receiving CMDF. Abdominal distension was the most frequently documented event, followed by increased gastric residuals, vomiting, suspected cow’s milk protein allergy, blood in stool, necrotizing enterocolitis (NEC), diarrhea, and other gastrointestinal symptoms. Some infants had more than one documented adverse feeding event; therefore, the sum of individual event categories exceeded the total number of infants with adverse events (n = 315).

Abdominal distension was the most frequently reported adverse event, followed by increased gastric residuals, vomiting, and suspected cow’s milk protein allergy. Necrotizing enterocolitis was uncommon, occurring in only six infants (Figure 1).

### 3.4. Adverse Feeding Events According to Maturity

**Figure 2:**
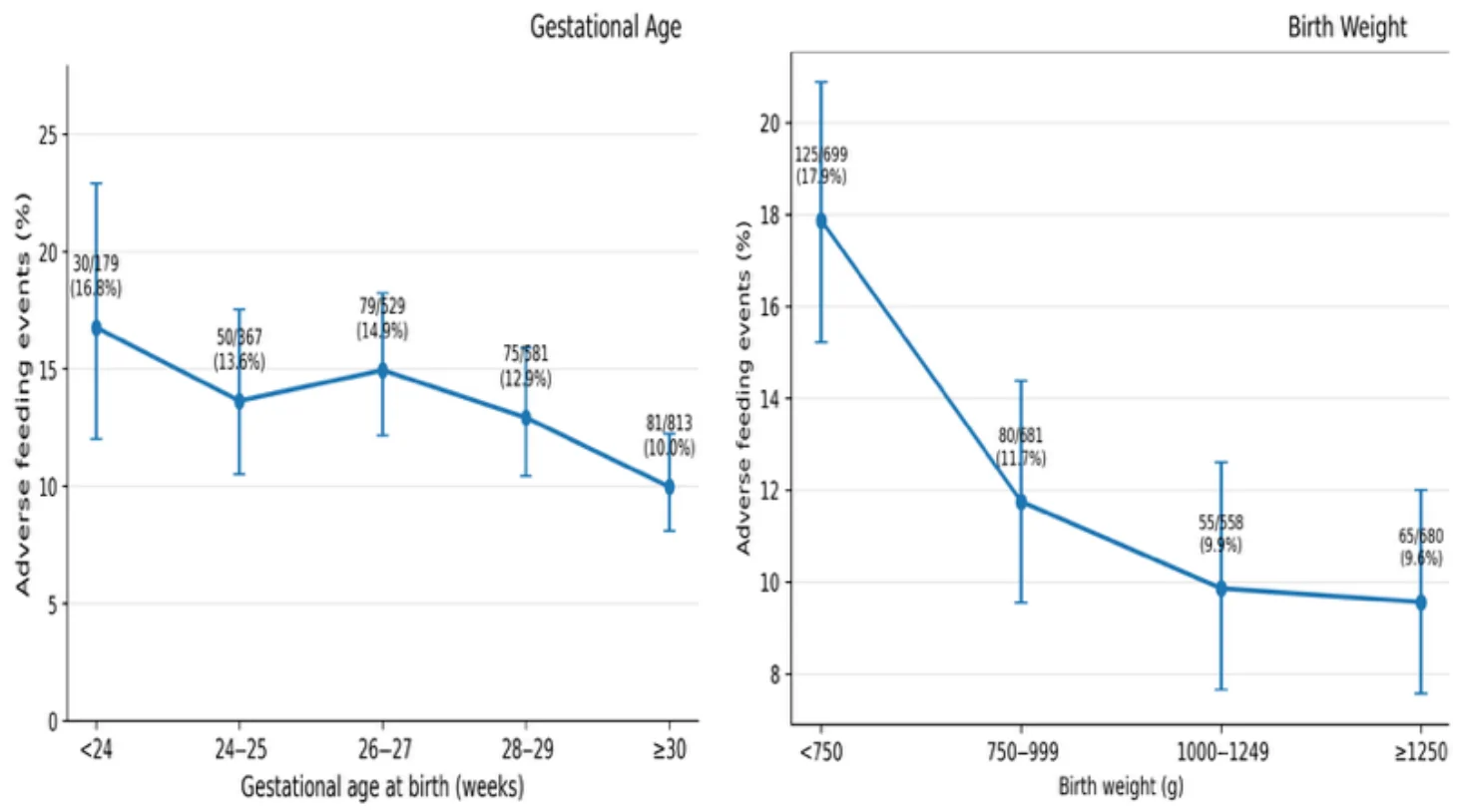
The incidence of documented adverse feeding events was generally higher among infants with lower gestational age and birth weight. Infants born before 24 weeks of gestation and those with a birth weight < 750 g had the highest incidence of documented adverse feeding events. Error bars indicate 95% confidence intervals.

The incidence of adverse feeding events increased with decreasing gestational age and birth weight (Table 3, Figure 2). Infants born before 24 weeks of gestation and those weighing < 750 g at birth demonstrated the highest incidence of adverse feeding events.

### 3.5. Multivariable Analysis

In multivariable logistic regression analysis, a longer time to achieve an enteral feeding volume of 100 mL/kg/day remained independently associated with documented adverse feeding events. In contrast, gestational age, birth weight, and sex were not independently associated with documented adverse feeding events after adjustment for the variables included in the model (Table 4). In a sensitivity analysis additionally adjusting for calendar year, calendar year was not independently associated with documented adverse feeding events (adjusted OR 1.067 per year, 95% CI 0.990–1.150; *p* = 0.090), whereas delayed achievement of an enteral feeding volume of 100 mL/kg/day remained independently associated with documented adverse feeding events (adjusted OR 1.020 per 1-day increase, 95% CI 1.005–1.036; *p* = 0.009).

**Figure 3:**
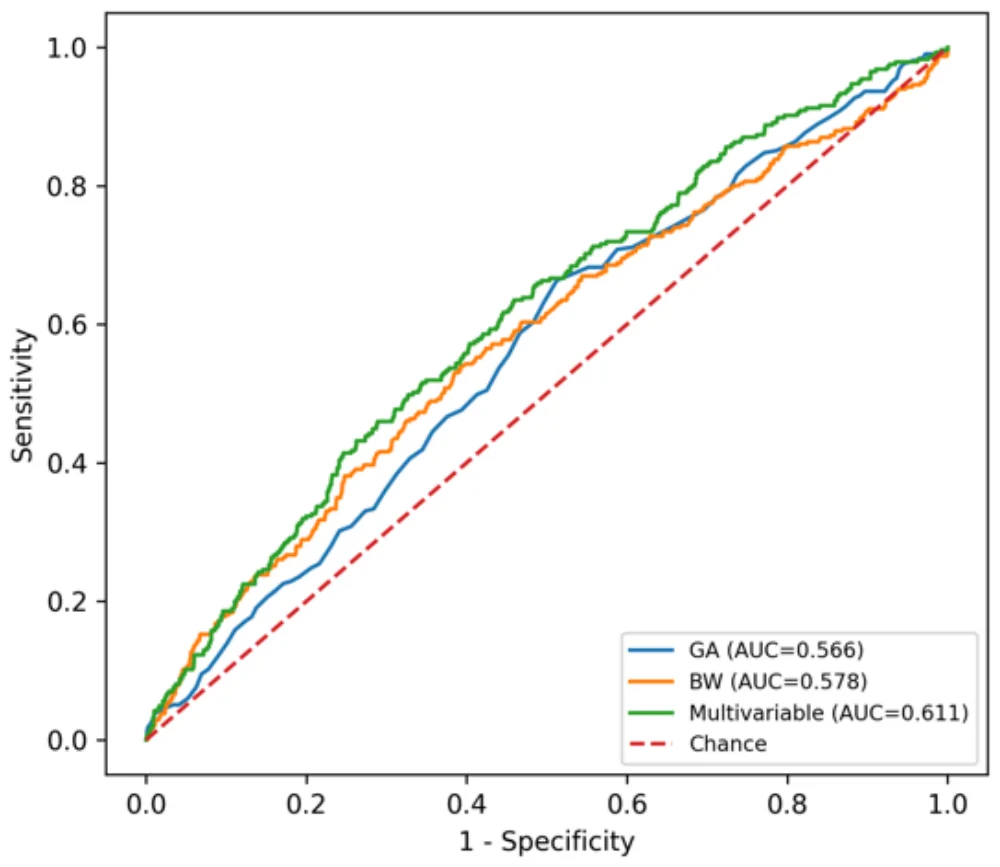
Receiver operating characteristic (ROC) analysis for the prediction of adverse feeding events. Receiver operating characteristic curves showing the predictive performance of gestational age, birth weight, the day reaching an enteral feeding volume of 100 mL/kg/day, and the multivariable logistic regression model. Routine clinical variables demonstrated only modest discriminatory ability, with the multivariable model showing the highest predictive performance (AUC = 0.611).

Receiver operating characteristic analysis demonstrated that routinely available clinical variables had only modest discriminatory ability for predicting adverse feeding events, with the multivariable model yielding an area under the curve of 0.611 (Figure 3).

## 4. Discussion

The present nationwide study yielded three principal findings. First, most infants receiving CMDF had no documented adverse feeding events. Second, in unadjusted analyses, documented adverse feeding events were more frequent among infants with lower gestational age and birth weight. Third, after adjustment for the variables included in the multivariable model, delayed achievement of an enteral feeding volume of 100 mL/kg/day remained associated with documented adverse feeding events, whereas gestational age, birth weight, and sex were not independently associated with these events.

The association between delayed achievement of 100 mL/kg/day and documented adverse feeding events should be interpreted cautiously. Time to reach this feeding milestone is a nonspecific measure of enteral feeding progression and may reflect multiple aspects of an infant’s clinical course, including underlying illness, complications of prematurity, fetal growth, feeding practices, and institutional management. Therefore, our findings do not establish that delayed feeding progression represents gastrointestinal immaturity or predicts intolerance to CMDF. Rather, they indicate that slower enteral feeding progression and documented adverse feeding events tended to occur in the same infants. Similarly, the absence of independent associations with gestational age and birth weight after multivariable adjustment should not be interpreted as evidence that biological maturity is unimportant, because residual confounding by clinical factors not captured in the database cannot be excluded.

An important consideration is that the adverse feeding events examined in this study were nonspecific. Abdominal distension, increased gastric residuals, vomiting, bloody stool, and related symptoms may occur for multiple reasons in preterm infants, including gastrointestinal immaturity, underlying illness, medications, and other complications of prematurity. Thus, the present findings should not be interpreted as demonstrating that CMDF caused these events. Rather, this study describes clinical characteristics associated with documented feeding difficulties among infants receiving CMDF in routine neonatal practice.

Although NEC represents a substantially more serious neonatal morbidity, the feeding events examined here address a different clinical issue. NEC was uncommon in the present cohort, occurring in only six infants. Less severe feeding difficulties nevertheless remain clinically relevant because recurrent abdominal distension, increased gastric residuals, vomiting, and related symptoms may interrupt or delay advancement of enteral feeding and complicate nutritional management. The present study was therefore not intended to evaluate the established relationship between bovine-derived nutritional products and NEC, but rather to characterize documented feeding events among infants receiving CMDF.

Our findings do not suggest that CMDF should be avoided. On the contrary, most infants receiving CMDF had no documented adverse feeding events. Exclusive human milk diets (EHMDs) have been evaluated in relation to major neonatal outcomes, including NEC and other morbidities, and these outcomes provide the principal rationale for considering EHMD in selected high-risk infants [22,23,24]. However, the present study did not directly compare CMDF with human milk-derived fortifiers and therefore cannot determine whether infants with delayed feeding progression or documented feeding difficulties would benefit from an EHMD.

A small case series by Sandhu et al. described very-low-birth-weight infants with recurrent gastrointestinal intolerance during CMDF use who subsequently tolerated human milk-derived fortifier as rescue therapy [25]. Although those observations were based on a very small uncontrolled series, they support prospective evaluation of alternative fortification strategies in selected infants with recurrent feeding difficulties.

The present findings should also be interpreted within the broader context of breastfeeding support and access to neonatal nutritional resources. CMDF use does not replace MOM or diminish the importance of breastfeeding support; rather, fortification addresses the nutritional gap between unfortified human milk and the high nutrient requirements of very preterm infants. The clinically relevant question is therefore not whether human milk or fortification should be prioritized, but how human milk can be fortified appropriately for an individual infant while preserving MOM whenever possible.

Access to human milk-derived fortifiers and EHMD may also depend on institutional resources, product availability, donor milk supply, reimbursement, and healthcare financing. These factors may contribute to center-level or healthcare-system differences in access. Because our database did not include individual socioeconomic information, the present study could not determine whether access differed according to parental socioeconomic status or social class. Future studies incorporating both clinical and health-system variables may help determine whether access to different fortification strategies is equitable.

Finally, routinely available clinical variables demonstrated only modest discriminatory ability for documented adverse feeding events. This finding indicates that gestational age, birth weight, sex, and feeding progression alone are insufficient for reliable clinical discrimination. The present model should therefore not be used for clinical prediction or the selection of fortification strategy. Instead, the observed associations should be considered hypothesis-generating and may help inform the design of prospective studies incorporating more detailed measures of illness severity, feeding trajectory, gastrointestinal function, fetal growth, and the precise timing of fortifier exposure and adverse events.

## 5. Limitations

This study has several limitations. First, the exact timing, dose, and formulation of CMDF initiation were unavailable in the database. Consequently, the temporal relationship among CMDF initiation, achievement of an enteral feeding volume of 100 mL/kg/day, and individual adverse feeding events could not be established. The day of achieving 100 mL/kg/day was therefore evaluated only as a pragmatic measure of enteral feeding progression and should not be interpreted as a surrogate for the timing of CMDF initiation or as a validated marker of gastrointestinal maturation. Future prospective studies should record the exact timing, dose, and type of fortifier exposure and directly link these data to the onset and resolution of gastrointestinal symptoms.

Second, adverse feeding events were identified from database records and may have been influenced by inter-institutional variation in clinical judgment and reporting. NEC was recorded as stage 2 or higher; however, detailed operational definitions and severity criteria were not uniformly available for several other adverse feeding events, including quantitative thresholds for gastric residuals or abdominal distension, or whether blood in the stool was occult or gross. Future studies should use prospectively defined criteria, provide standardized training to participating healthcare professionals, and consider central adjudication or independent review of a subset of events.

Third, routinely available clinical variables demonstrated only modest discriminatory ability. Important potential confounders, including detailed measures of illness severity, medication exposure, respiratory support, and complications of prematurity, were not consistently available or sufficiently standardized in the database. Future prospective studies should incorporate these variables, together with detailed feeding trajectories and potentially relevant biomarkers, to develop and externally validate more informative models.

Fourth, although additional adjustment for calendar year did not materially alter the principal findings, calendar year may not have fully captured the pandemic-related changes in healthcare delivery. The database did not include detailed information regarding institution-specific changes in staffing, feeding protocols, or NICU practices during the COVID-19 pandemic. Future studies should prospectively account for temporal and institution-level changes in neonatal care.

Fifth, SGA and fetal growth restriction were not directly recorded in the database and therefore could not be included as covariates. Although gestational age and birth weight were included separately in the multivariable model, these variables do not fully capture fetal growth relative to gestational age. Residual confounding related to fetal growth therefore cannot be excluded. Future prospective studies should systematically record standardized measures of fetal growth and incorporate SGA status into multivariable analyses.

Finally, adverse-event information was unavailable for 111 infants. Because the missing variable was the primary outcome, multiple imputation was not performed, as reliable imputation would have required assumptions regarding the missingness mechanism that could not be verified from the available data. Future prospective database studies should incorporate mandatory outcome fields, automated completeness checks, and real-time data validation to minimize missing outcome information.

## 6. Conclusions

Most preterm infants receiving CMDF had no documented adverse feeding events. Among the CMDF-exposed infants, delayed achievement of an enteral feeding volume of 100 mL/kg/day was associated with documented adverse feeding events; however, the temporal relationship among CMDF initiation, feeding progression, and adverse events could not be established from the database. Therefore, this finding should be considered hypothesis-generating rather than predictive. Prospective studies capturing the exact timing of CMDF initiation, feeding progression, and adverse events are needed to determine whether feeding trajectory can contribute to individualized fortification strategies.

## Figures and Tables

**Table 2:** Baseline characteristics of CMDF-exposed infants with and without documented adverse feeding events.

Variable	No AE n	No AE Median (IQR)	AE n	AE Median (IQR)	*p* Value
Gestational age, weeks	2154	28.7 (26.3–30.9)	315	27.9 (25.9–30.0)	<0.001
Birth weight, g	2161	994.0 (753.0–1264.0)	315	872.0 (645.5–1189.0)	<0.001
Day reaching 100 mL/kg/day	1971	9.0 (7.0–12.0)	285	11.0 (8.0–15.0)	<0.001
Male sex	2160	1128 (52.2%)	315	159 (50.5%)	0.562

Values are presented as median (interquartile range) or n (%). Adverse feeding events were those documented in the database among infants receiving CMDF. Although these events were recorded in relation to CMDF use, the exact date of CMDF initiation was unavailable; therefore, the precise temporal relationship between CMDF initiation and individual adverse feeding events could not be established. AE, adverse feeding event; CMDF, cow’s milk-derived fortifier.

**Table 3:** Incidence of adverse feeding events according to gestational age and birth weight.

Gestational Age	Total, n (%)	AE, n (%)	Birth Weight	Total, n (%)	AE, n (%)
<24 wk	179 (7.2)	30 (16.8)	<750 g	656 (26.5)	120 (18.3)
24–25 wk	367 (14.9)	50 (13.6)	750–999 g	650 (26.3)	78 (12.0)
26–27 wk	529 (21.4)	79 (14.9)	1000–1249 g	538 (21.7)	54 (10.0)
28–29 wk	581 (23.5)	75 (12.9)	≥1250 g	632 (25.5)	63 (10.0)
≥30 wk	813 (32.9)	81 (10.0)			

Values are presented as n (%). Percentages in the total columns represent the proportion of infants within each gestational-age or birth-weight category; percentages in the AE columns represent the proportion of infants with documented adverse feeding events within each category. Gestational age was available for 2469 infants and birth weight for 2476 infants. AE, adverse feeding event.

**Table 4:** Multivariable logistic regression analysis of documented adverse feeding events among infants receiving CMDF.

Variable	Adjusted OR	95% CI	*p* Value
Gestational age (per 1-week increase)	0.974	0.911–1.041	0.439
Birth weight (per 100-g increase)	0.949	0.892–1.011	0.104
Male sex	0.934	0.726–1.203	0.598
Days to reach 100 mL/kg/day (per 1-day increase)	1.021	1.006–1.037	0.007

## Data Availability

The data presented in this study are available from the corresponding author upon reasonable request.
